# Supplementary material for: Quantifying telomeric lncRNAs using PNA-labelled RNA-Flow FISH (RNA-Flow)
Source: Commun Biol. 2022 May 25;5:513. doi: 10.1038/s42003-022-03452-3 (PMC9132901; doi:10.1038/s42003-022-03452-3)
Supplement: Supplementary file 2 — Supplementary information [file 42003_2022_3452_MOESM2_ESM.pdf]

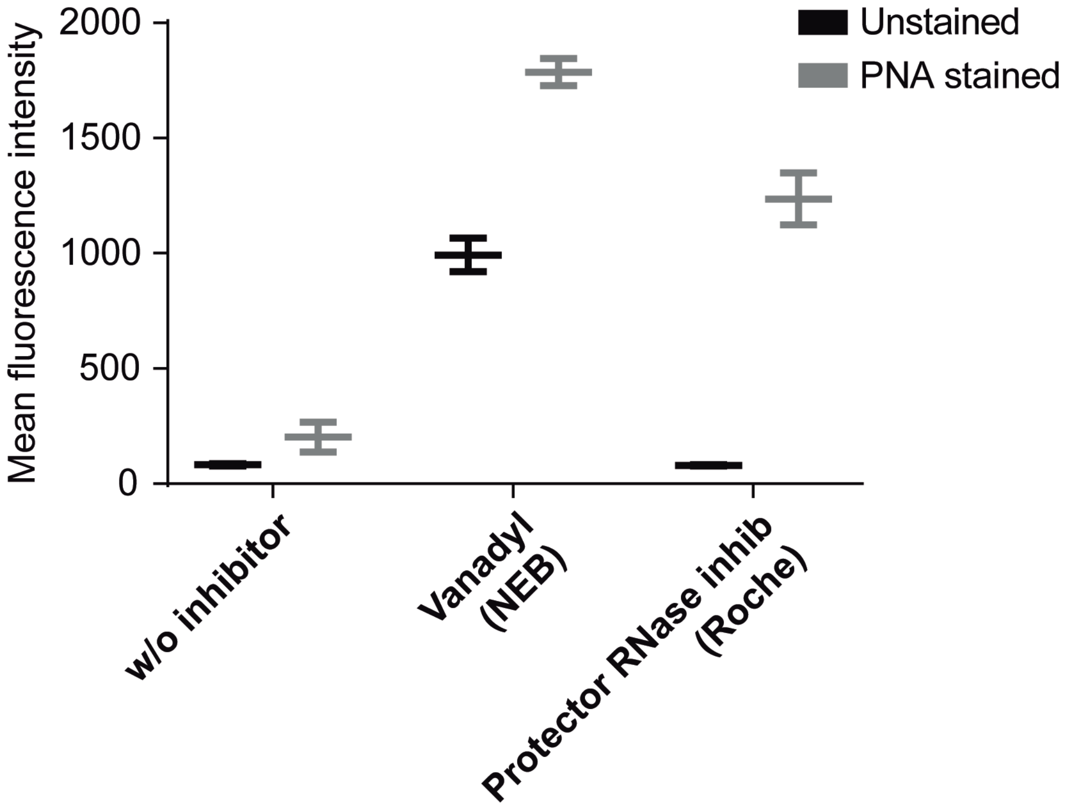

Supplementary figure 1: RNase inhibitor study. Two protectors RNase inhibitors were tested to find a suitable one for the assay. Vanadyl form NEB was discarded due to autofluorescence whilst the RNase inhibitor from Roche (03335399001) was suitable to protect the RNA and increase the signal without interfering with the methodology used.

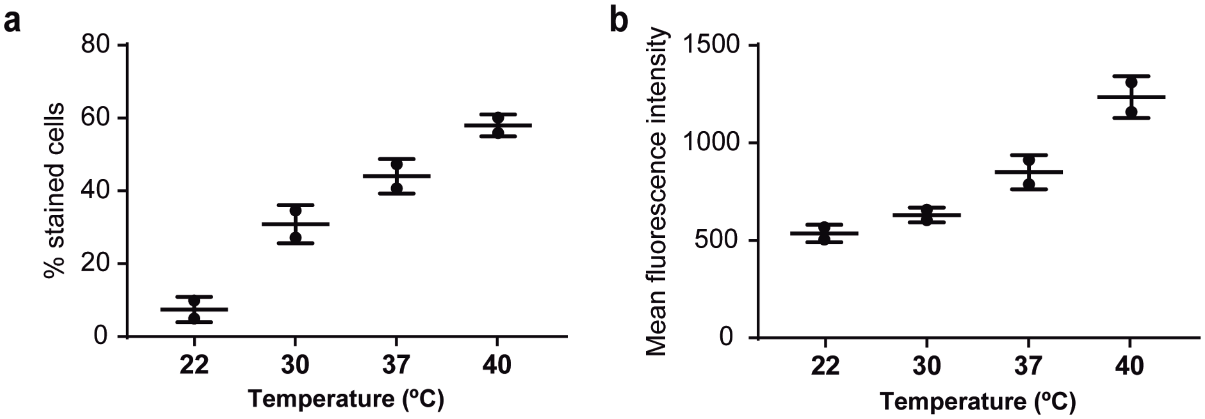

Supplementary figure 2: Temperature study. Temperatures ranging from room temperature to 40ºC were tested in order to establish the best assay conditions. a) regarding number of cells stained and b) mean fluorescence intensity detected. It was also important to make sure the reduction of the melting point due to the formamide in the hybridisation buffer did not infer cross reactions with the DNA.

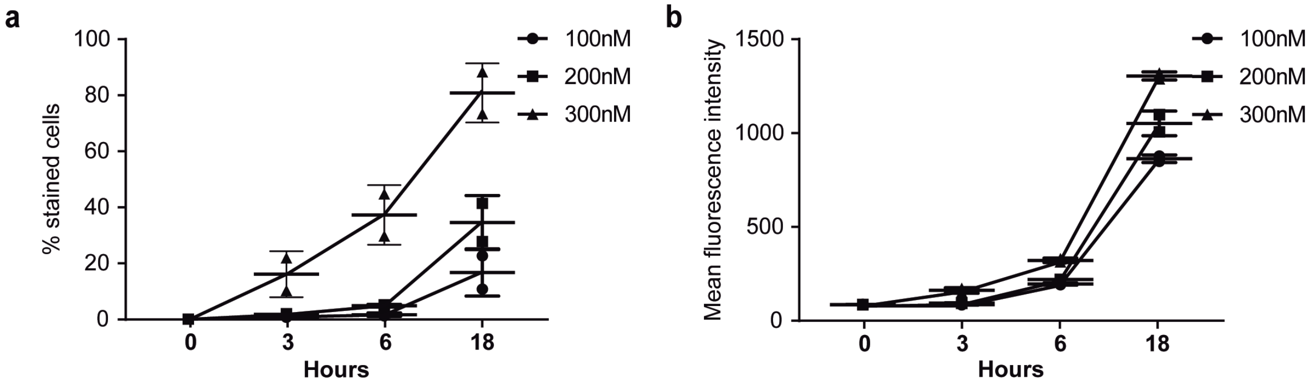

Supplementary figure 3: PNA hybridization condition study for RNA staining. Curves depicted with the different assay conditions tested, time and concentration wise**.** a) percentage of cells stained at different probe concentrations and at different times after addition of the probe. b) Mean RNA fluorescence intensity of cells stained with different probe concentrations and at different times after the addition of the probe

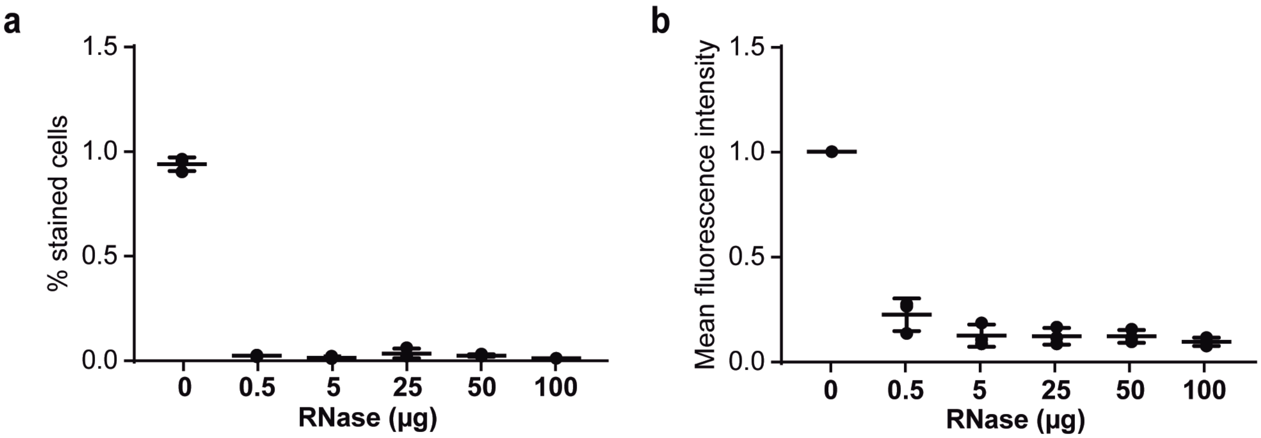

Supplementary figure 4: RNase study. Incubating the cells with the PNA probe and different RNA concentrations allowed us to demonstrate that with 0.5 µg we could almost abolish the PNA signal in terms of a) number of cells stained and b) mean fluorescence intensity per sample.

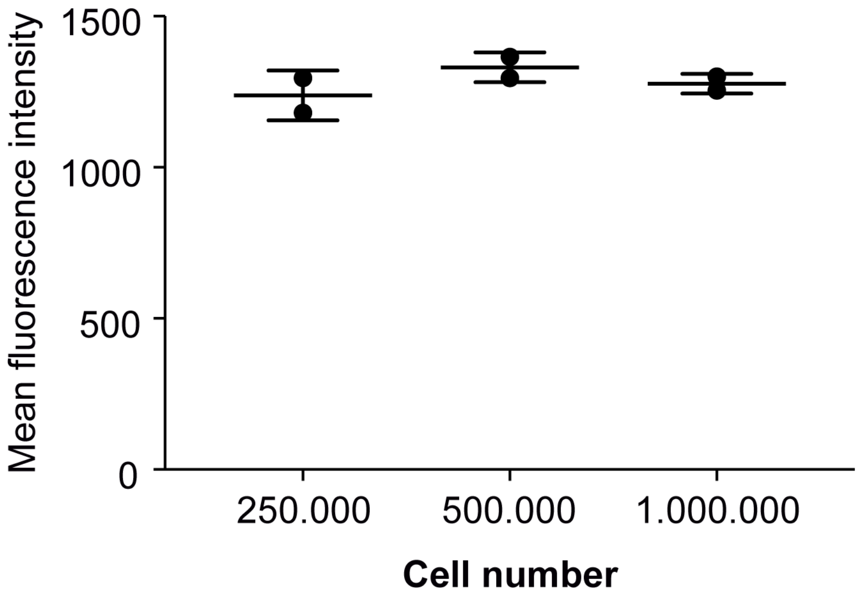

Supplementary figure 5: Cell number study. Cell number trial to determine whether or not the cell number is critical for the assay. Between 0.25*10^6; 0.5*10^6; and 1*10^6 cells the assay can be performed without significantly affecting the signal intensity or the number of labelled cells.

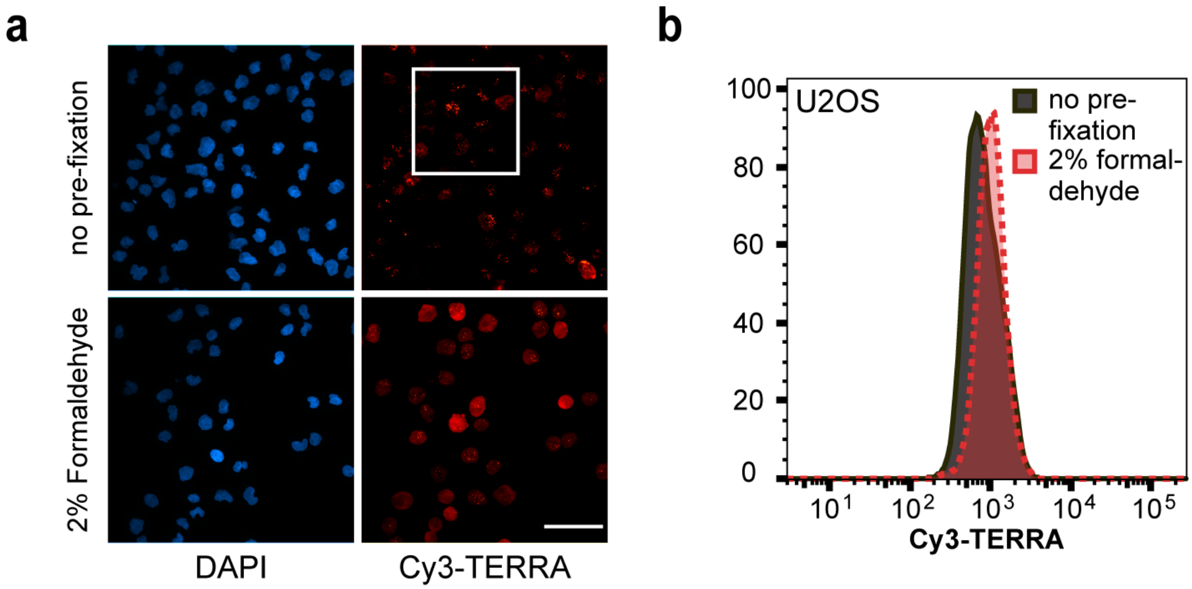

Supplementary figure 6: Pre-fixation study for TERRA staining. a) Confocal images of U2OS of TERRA staining using the RNA-Flow staining protocol. Left panels are the DAPI staining showing nuclear integrity and the right panels show the TERRA staining with Cy3-PNA probe anti-TERRA lncRNA. The upper panels were stained with no previous pre-fixation and lower panels were pre-fixed with 2% formaldehyde. Z-stacks of 80 images were captured in 0.47 µm sections across the entire nucleus with a Nikon A1R laser scanning confocal microscope (Nikon) using a 60X/1.4 Plan-Apochromat objective. Please note, the white square on the Cy3-TERRA upper panel shows the region of interest (ROI) used in figure 1C. Scale bar is 50 µm. b) RNA-Flow of the same U2OS cells for TERRA staining analysed by flow cytometry. Black peak shows Cy3-Terra staining with no pre-fixation and the red peak shows the fluorescence intensity of the staining after a pre fixation with 2% formaldehyde.

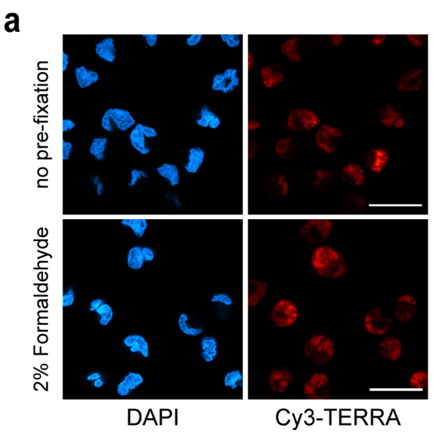

b

TERC Sequence (human):

GGGTTGCGGAGGGTGGGCCTGGGAGGGGTGGTGGCCATTTTTTGTCTAACCCTAACTGAGAAGGGCGTAGGCGCCGTGCTTT
TGCTCCCCGCGCGCTGTTTTTCTCGCTGACTTTCAGCGGGCGGAAAAGCCTCGGCCTGCCGCCTTCCACCGTTCATTCTAGAG
CAAACAAAAAATGTC***AGCTGCTG**GCCCGTTCGCCCCTCCCGGGGACCTGCGGCGGGTCGCCTGCCCAGCCCCCGAACCCC
GCCTGGAGGCCGCGGTCGGCCCGGGGCTTCTCCGGAGGCACCCACTGCCACCGCGAAGAGTTGGGCTCTGTCAGCCGCGGGT
CTCTCGGGGGCGAGGGCGAGGTTCAGGCCTTTCAGGCCGCAGGAAGAGGAACGGAGCGAGTCCCCGCGCGCGGCGCGATTCC
CTGAGCTGTGGGACGTGCACCCAGGACTCGGCTCACACATGCAGTTCGCTTTCCTGTTGGTGGGGGGAACGCCGATCGTGCGC
ATCCGTCACCCCTCGCCGGCAATGGGGGCTTGTGAACCCCCAAACCTG

PNA Sequence (Bold sequence): Cy3-OO-agcagctgacattttttgtttg.

Antisense GapmeR hTERC (Quiagen)***

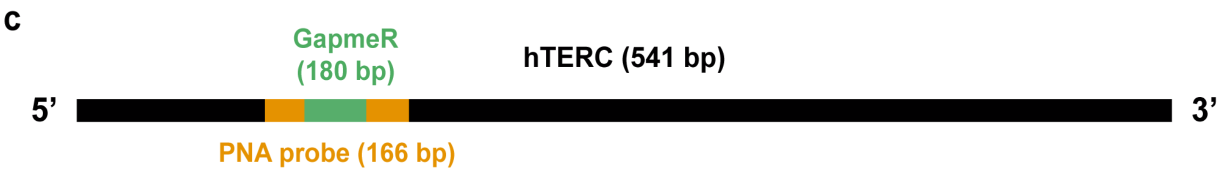

Supplementary figure 7: TERC probe details a) Fixation study. Confocal images of MDA-MB231 after TERC staining using the RNA-Flow staining protocol. Left panels are the DAPI staining showing nuclear integrity and the right panels show the TERC staining with a specific Cy3-PNA probe. The upper panels were stained with no previous pre-fixation and lower panels were pre-fixed with 2% formaldehyde. Z-stacks of 80 images were captured in 0.47 µm sections across the entire nucleus with a Nikon A1R laser scanning confocal microscope (Nikon) using a 60X/1.4 Plan-Apochromat objective. Scale bar is 25 µm. b) TERC sequence with indications for the PNA probe location and GapmeR location within the TERC sequence. c) Scheme with the positions of the probe and the GapmeR inside the human TERC sequence.

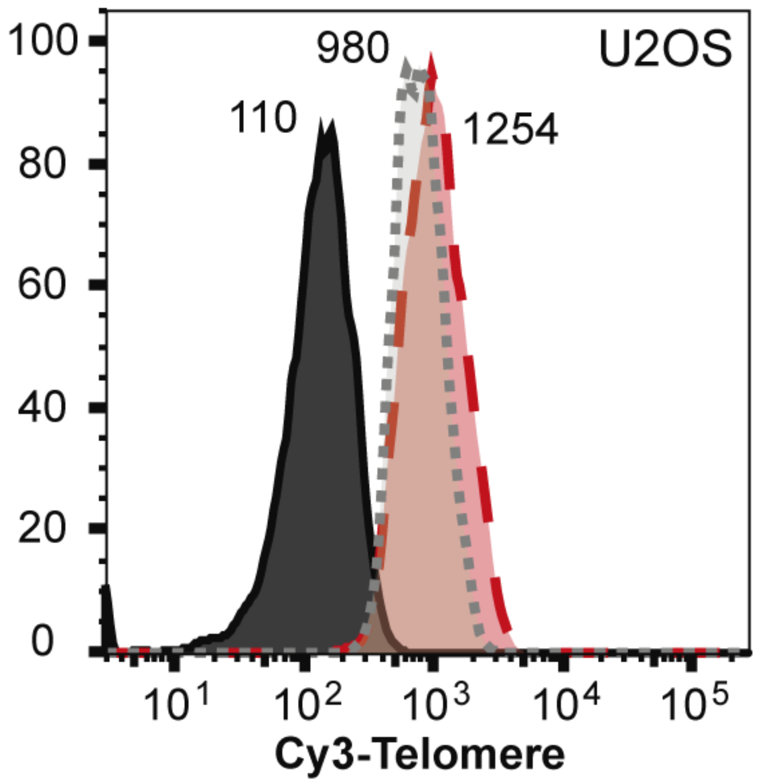

Supplementary figure 8: Telomere staining in U2OS with the TelC probe. The protocol was initiated with a denaturation for 10 minutes at 85ºC. Black peak represents the unstained cells. Red dashed line peak depicts the mean fluorescence of telomeres. Short dashed peak filled in grey is the stained cells with the RNase treatment. Note that in this case, the RNase treatment does not affect the staining as DNA is being labelled in this case.

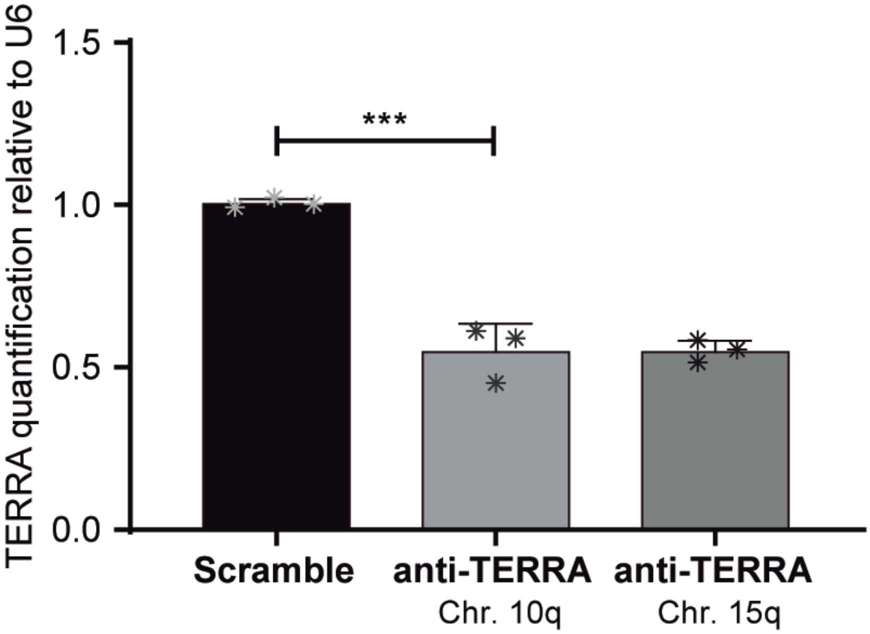

Supplementary figure 9: qPCR on GapmeR transfected U2OS cells on chromosomes 10q and 15q. Demonstration of the TERRA reduction after knockdown with the GapmeR technology 24 hours after transfection. *** $P \leq 0.0001$ using Student's t-test.

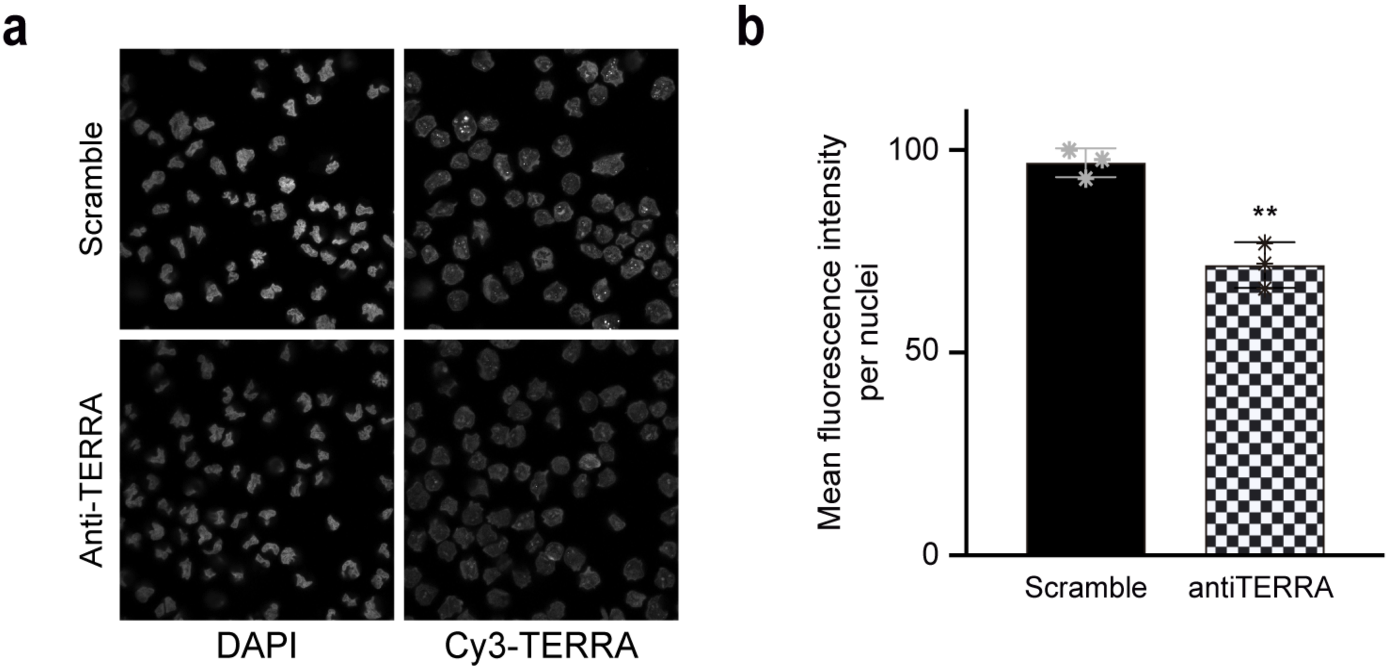

Supplementary figure 10: Confocal images of the transfected U2OS with either the scramble GapmeR or the anti-TERRA GapmeR after staining with the RNA-Flow protocol. a) Left panels are the DAPI staining and the right panels show the TERRA staining with Cy3-PNA probe anti-TERRA lncRNA in an specific image of the Z-stack lot obtained. Image is the 25th out of 80 for both samples, scramble (control) and anti-TERRA. b) Quantification of the confocal images of the transfected cells. Z-stacks of 80 images were captured in 0.47 µm sections across the entire nucleus with a Nikon A1R laser scanning confocal microscope (Nikon) using a 60X/1.4 Plan-Apochromat objective. Acquired Z-stacks were reconstructed (sum intensity projection) and analysed using Fiji v1.53.

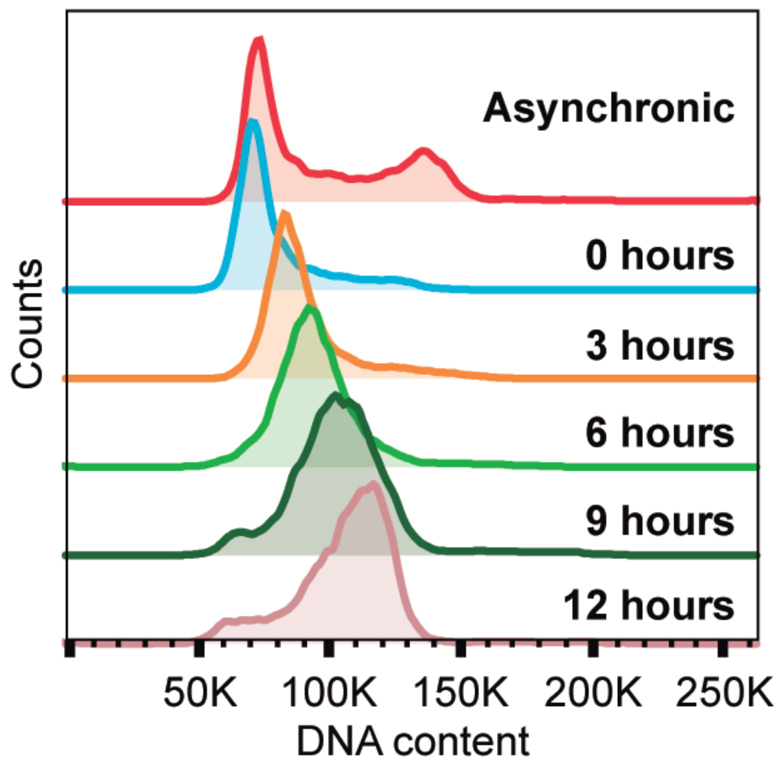

Supplementary figure 11: Time lapse of the cell cycle distribution over time after 12 hours of HU block in U2OS cells at 0,3,6,9 and 12 hours after block release.

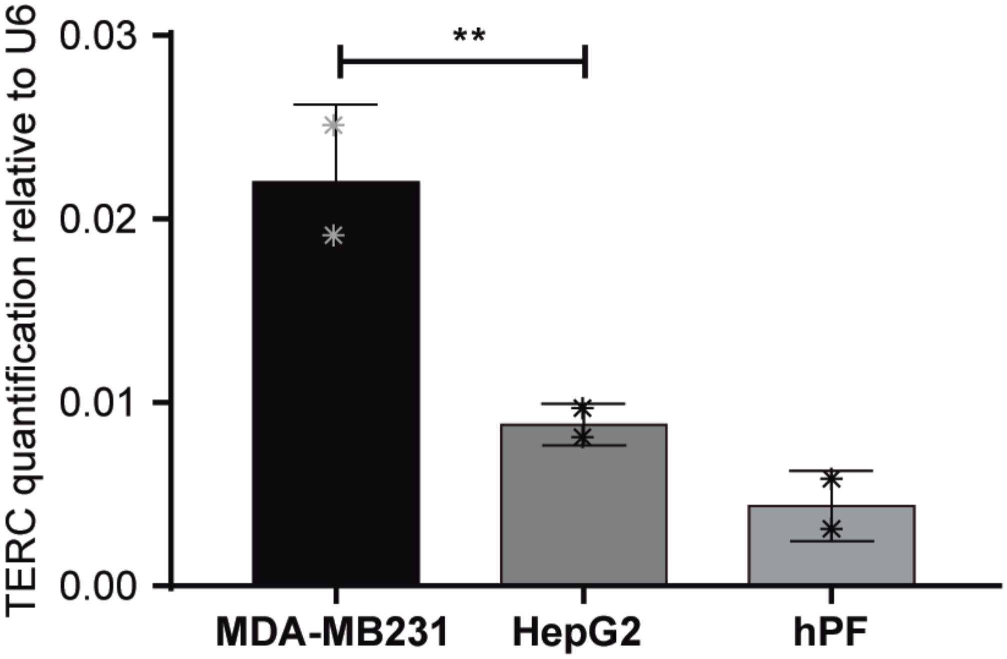

Supplementary figure 12: TERC study by qPCR of MDA-MB231, HepG2 and hPF cells. Results support those obtained using RNA-Flow. ** P ≤ 0.001 using Student's t-test. Pearson correlation index between the qPCR and the RNA-Flow: r = 0.9740, R^2 = 0.9487 and a p-value of 0.0001 (****).

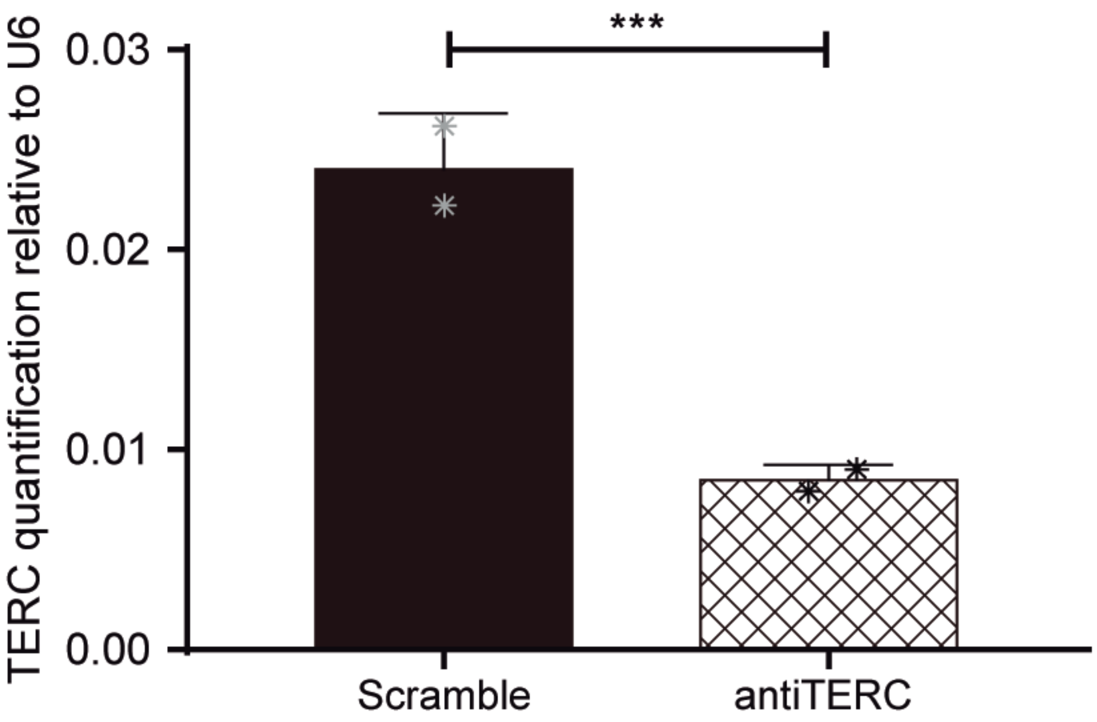

Supplementary figure 13: TERC study on knockdown MDA-MB231 by qPCR. Solid bar is the scramble control. Checked bar is the value of the TERC expression 24 hours after TERC GapmeR knockdown. *** $P \leq 0.0001$ using Student's t-test.

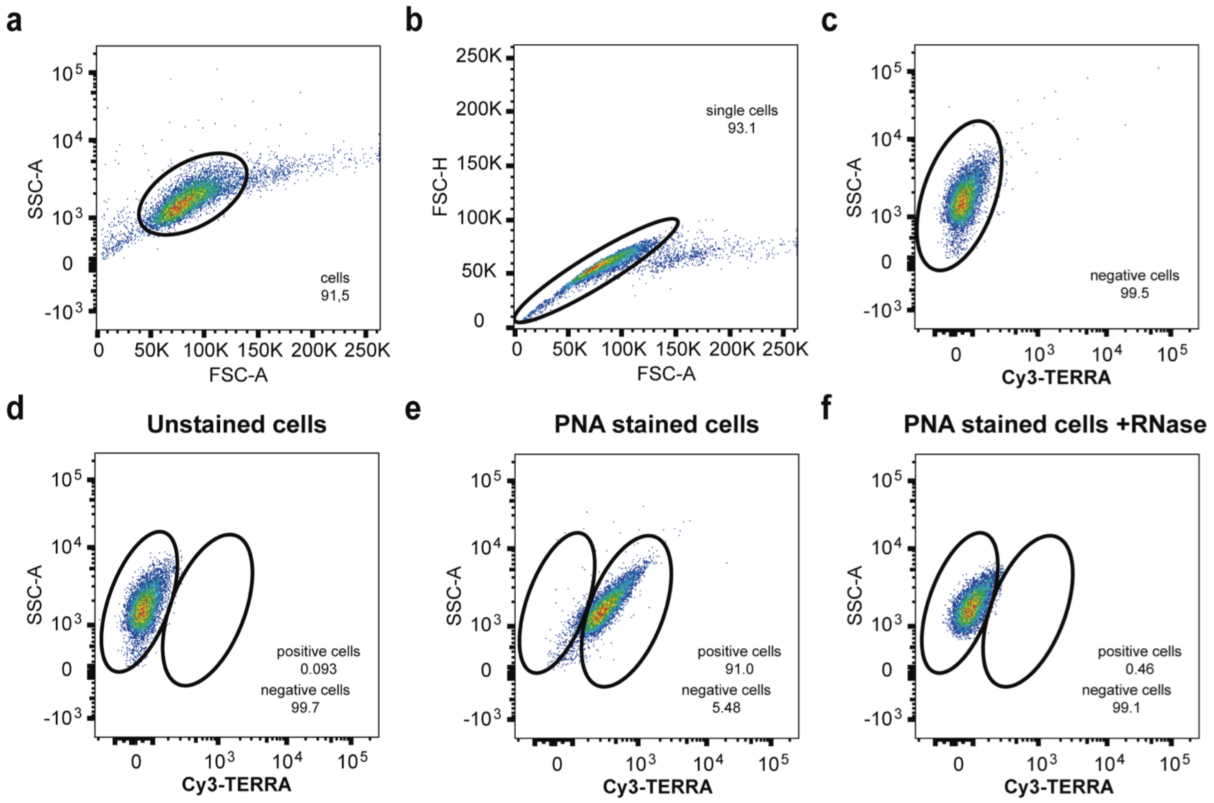

Supplementary figure 14: Cytometry approach to populations. a) Alive cells were gated on the SSC-A/FSC-A plot. b) Single cells were gated in the FSC-H/FSC-A plot. c) Negative cells were gated in the SSC-A/FL2 detector. d) Gating of the unstained cells. e) Gating of positive stained Cy3-TERRA samples. f) Gating of the PNA stained cells co-incubated with RNase H.
