# Supplementary material for: Quantifying telomeric lncRNAs using PNA-labelled RNA-Flow FISH (RNA-Flow)
Source: Commun Biol. 2022 May 25;5:513. doi: 10.1038/s42003-022-03452-3 (PMC9132901; doi:10.1038/s42003-022-03452-3)
Supplement: Supplementary file 5 — Reporting Summary [file 42003_2022_3452_MOESM5_ESM.pdf]

nature portfolio

Corresponding author(s): Dr Iria Gonzalez Vasconcellos

Last updated by author(s): Apr 21, 2022

Reporting Summary

Nature Portfolio wishes to improve the reproducibility of the work that we publish. This form provides structure for consistency and transparency in reporting. For further information on Nature Portfolio policies, see our Editorial Policies and the Editorial Policy Checklist.

Statistics

For all statistical analyses, confirm that the following items are present in the figure legend, table legend, main text, or Methods section.

n/a	Confirmed	
☐	☒	The exact sample size (*n*) for each experimental group/condition, given as a discrete number and unit of measurement
☐	☒	A statement on whether measurements were taken from distinct samples or whether the same sample was measured repeatedly
☐	☒	The statistical test(s) used AND whether they are one- or two-sided<br>*Only common tests should be described solely by name; describe more complex techniques in the Methods section.*
☐	☒	A description of all covariates tested
☐	☒	A description of any assumptions or corrections, such as tests of normality and adjustment for multiple comparisons
☐	☒	A full description of the statistical parameters including central tendency (e.g. means) or other basic estimates (e.g. regression coefficient) AND variation (e.g. standard deviation) or associated estimates of uncertainty (e.g. confidence intervals)
☐	☒	For null hypothesis testing, the test statistic (e.g. *F*, *t*, *r*) with confidence intervals, effect sizes, degrees of freedom and *P* value noted<br>*Give P values as exact values whenever suitable.*
☒	☐	For Bayesian analysis, information on the choice of priors and Markov chain Monte Carlo settings
☒	☐	For hierarchical and complex designs, identification of the appropriate level for tests and full reporting of outcomes
☐	☒	Estimates of effect sizes (e.g. Cohen's *d*, Pearson's *r*), indicating how they were calculated

Our web collection on statistics for biologists contains articles on many of the points above.

Software and code

Policy information about availability of computer code

Data collection	All the data collection in this manuscript was performed with the FACS Canto A using the BD FACSDiva software v8.0.1
Data analysis	The data analysis was performed using the FlowJo_v10.7.1 software. Statistical analyses (Student´s T-tests and Pearson correlation analyses) were performed using the GraphPad Prism v9 (GraphPad software Inc, California, USA). Analysis were performed in triplicates and the significance was represented using the p-value as follows: (*) p ≤0.05; (**) p ≤0.01; (***) p ≤0.001; (****) p ≤0.0001.

For manuscripts utilizing custom algorithms or software that are central to the research but not yet described in published literature, software must be made available to editors and reviewers. We strongly encourage code deposition in a community repository (e.g. GitHub). See the Nature Portfolio guidelines for submitting code & software for further information.

Data

Policy information about availability of data

All manuscripts must include a data availability statement. This statement should provide the following information, where applicable:

- Accession codes, unique identifiers, or web links for publicly available datasets
- A description of any restrictions on data availability
- For clinical datasets or third party data, please ensure that the statement adheres to our policy

Data sharing not applicable to this article as no datasets were generated or analysed during the current study.

Field-specific reporting

Please select the one below that is the best fit for your research. If you are not sure, read the appropriate sections before making your selection.

☒ Life sciences ☐ Behavioural & social sciences ☐ Ecological, evolutionary & environmental sciences

For a reference copy of the document with all sections, see nature.com/documents/nr-reporting-summary-flat.pdf

Life sciences study design

All studies must disclose on these points even when the disclosure is negative.

Sample size	We initially performed a trial experiment to demonstrate reproducibility. We found out that N=3 was accurate enough for a proper mean and standard deviation value. This is why we chose to repeat the presented experiments 3 times. Technical reproducibility was statisically significant and biological reproducibility in the case of PBMCs was also mathematically accurate with an N=3.
Data exclusions	No data was excluded from our analysis except for that obtained in the first trial experiments to demonstrate reproducibility. Once the trial experiments were operational, all data presented here was repeated 3 times.
Replication	All attempts of replication (3) were successful.
Randomization	It does not apply to the study.
Blinding	It does not apply to the study.

Reporting for specific materials, systems and methods

We require information from authors about some types of materials, experimental systems and methods used in many studies. Here, indicate whether each material, system or method listed is relevant to your study. If you are not sure if a list item applies to your research, read the appropriate section before selecting a response.

Materials & experimental systems

n/a	Involved in the study	
☒	☐	Antibodies
☐	☒	Eukaryotic cell lines
☒	☐	Palaeontology and archaeology
☒	☐	Animals and other organisms
☒	☐	Human research participants
☒	☐	Clinical data
☒	☐	Dual use research of concern

Methods

n/a	Involved in the study	
☒	☐	ChIP-seq
☐	☒	Flow cytometry
☒	☐	MRI-based neuroimaging

Eukaryotic cell lines

Policy information about cell lines

Cell line source(s)	Hep G2 (ATCC Catalogue number HB-8065), MDA-MB-231 (ATCC Catalogue number HTB-26), U-2 OS (ATCC Catalogue number HTB-96). Human fibroblasts: Explanted from muscle from abortive tissue. The participant provided written informed consent in accordance with the Declaration of Helsinki.<br>PMBCs: Ficoll isolation from whole blood samples (Human donors). The participant provided written informed consent in accordance with the Declaration of Helsinki.
Authentication	No Authentication was performed.
Mycoplasma contamination	Cells tested negative in routine tests of mycoplasma contamination at our institution.
Commonly misidentified lines (See ICLAC register)	*Name any commonly misidentified cell lines used in the study and provide a rationale for their use.*

Flow Cytometry

Plots

Confirm that:

☒ The axis labels state the marker and fluorochrome used (e.g. CD4-FITC).

☒ The axis scales are clearly visible. Include numbers along axes only for bottom left plot of group (a 'group' is an analysis of identical markers).

☒ All plots are contour plots with outliers or pseudocolor plots.

☒ A numerical value for number of cells or percentage (with statistics) is provided.

Methodology

Sample preparation	All cell lines used in this study (U2OS, HEPG2, MDA-MB231 and hPF) were kept in culture in DMEM (Gibco) with 10% foetal bovine serum and 1% glutamine. Incubated at 37ºC in a humidified atmosphere with 5% CO2. PBMCs were obtained from whole blood samples. 10 ml of blood was diluted 1:2 in PBS and isolated via ficoll density gradient. The participants provided written informed consent in accordance with the Declaration of Helsinki.<br><br>0,5x106 cells were collected via trypsinization and washed in 500 µL of PBS. Cells were centrifuged at 260g for 5 minutes. Supernatant was discarded and cellular pellets were resuspended in 500 µL hybridisation buffer.
Instrument	BD FACS Canto Flow cytometerTM. Octubre 2005. Reference 337175. Serial number: V07300449
Software	FACS Canto A using the BD FACSDiva software v8.0.1<br>The data analysis was performed using the FlowJo_v10.7.1 software.
Cell population abundance	No sorting was performed.
Gating strategy	The gating strategy is shown on supplemetary figure 11 with an example of all the subsequent gates.

☒ Tick this box to confirm that a figure exemplifying the gating strategy is provided in the Supplementary Information.
